# The heating effect of iron-cobalt magnetic nanofluids in an alternating magnetic field: application in magnetic hyperthermia treatment

**DOI:** 10.1186/1556-276X-8-540

**Published:** 2013-12-20

**Authors:** Ali Shokuhfar, Seyyed Salman Seyyed Afghahi

**Affiliations:** 1 Advanced Materials and Nanotechnology Research Laboratory, Department of Materials Science and Engineering, K.N. Toosi University of Technology, P.O. Box 19395–1999, Tehran, Iran

**Keywords:** FeCo alloy nanoparticles, Hyperthermia treatment, Specific absorption rate, Microemulsion, Magnetic properties, Anisotropy

## Abstract

In this research, FeCo alloy magnetic nanofluids were prepared by reducing iron(III) chloride hexahydrate and cobalt(II) sulfate heptahydrate with sodium borohydride in a water/CTAB/hexanol reverse micelle system for application in magnetic hyperthermia treatment. X-ray diffraction, electron microscopy, selected area electron diffraction, and energy-dispersive analysis indicate the formation of bcc-structured iron-cobalt alloy. Magnetic property assessment of nanoparticles reveals that some samples are single-domain superparamagnetic, while others are single- or multi-domain ferromagnetic. The stability of the magnetic fluids was achieved by using a CTAB/1-butanol surfactant bilayer. Results of Gouy magnetic susceptibility balance experiments indicate good stability of FeCo nanoparticles even after dilution. The inductive properties of corresponding magnetic fluids including temperature rise and specific absorption rate were determined. Results show that with increasing of the nanoparticle size in the single-domain size regime, the generated heat increases, indicating the significant effect of the hysteresis loss. Finally, the central parameter controlling the specific absorption rate of nanoparticles was introduced, the experimental results were compared with those of the Stoner-Wohlfarth model and linear response theory, and the best sample for magnetic hyperthermia treatment was specified.

## Background

Magnetic nanoparticles are a topic of growing interest because of their versatile applications such as drug delivery, magnetic hyperthermia, magnetic separation, magnetic resonance imaging (MRI) contrast enhancement, and ultrahigh-density data storage [1-14]. Among those, magnetic hyperthermia is a novel therapeutic method in which the magnetic nanoparticles are subjected to an alternating magnetic field to generate a specific amount of heat to raise the temperature of a tumor to about 42°C to 46°C at which certain mechanisms of cell damage are activated [15,16].

These mechanisms which produce heat in alternating current (AC) magnetic fields include the following: (1) hysteresis, (2) Neel or Brownian relaxation, and (3) viscous losses [17]. The generated heat is quantitatively described by the specific absorption rate (SAR) of nanoparticles which is related to specific loss per cycle of hysteresis loop (*A*) by the equation SAR = *A* × *f* in which *f* is the frequency of the applied field.

There are four models based on size regimes to describe the magnetic properties of nanoparticles [17]:

1. At superparamagnetic size regime in which the hysteresis area is null, the equilibrium functions are used. In this size range depending on the anisotropy energy, the magnetic behavior of nanoparticles progressively changes from the Langevin function (*L*(*ξ*) = coth(*ξ*) - 1/*ξ*) for zero anisotropy to tanh(*ξ*) for maximal anisotropy where *ξ* = (*μ*_0_*M*_*s*_*VH*_max_)/(*k*_B_*T*).

2. Around the superparamagnetic-ferromagnetic transition size, the linear response theory (LRT) does the job for us. The LRT is a model for describing the dynamic magnetic properties of an assembly of nanoparticles using the Neel-Brown relaxation time and assumes a linear relation between magnetization and applied magnetic field. The area of the hysteresis loop is determined by [17]

(1) $$A=\frac{\pi \mu_{0}^{2}H_{\max}^{2}M_{s}^{2}V}{3k_{B}T}\frac{\omega \tau_{R}}{\left(1+\omega^{2}\tau_{R}^{2}\right)}\left(3-\frac{2}{1+{\left(\frac{\sigma }{3.4}\right)}^{1.47}}\right)$$

where *σ* = KV/*k*_B_*T*, *ω* = *2*π*f*, and *τ*_R_ is the relaxation time of magnetization which is assumed to be equal to the Neel-Brown relaxation time (*τ*_N_).

3. In the single-domain ferromagnetic size regime, the Stoner-Wohlfarth (SW)-based models are applied which neglect thermal activation and assume a square hysteresis area that is practically valid only for *T* = 0 K or *f* → *∞* but indicates the general features of the expected properties for other conditions.

Based on the SW model for magnetic nanoparticles with their easy axes randomly oriented in space, the hysteresis area is calculated by [17]

(2) $$A=2\mu_{0}H_{c}M_{s}$$

4. Finally, for multi-domain ferromagnetic nanoparticles, there is no simple way to model the magnetic properties of such large nanoparticles. In hyperthermia experiments, increasing the nanoparticle size to multi-domain range promotes the probability of precipitation of nanoparticles which leads to the blockage of blood vessels. Also, the larger the nanoparticle size, the higher the chance for the phagocytosis system to detect it. So, this size range has not drawn considerable attention for use in hyperthermia treatment.

The key factor to obtain the maximum SAR in conventional clinical hyperthermia treatments (*f* = 120 kHz, *μ*_0_*H*_max_ =20 mT, *T* = 300 K) is the anisotropy of synthesized nanoparticles. Calculations of SAR as a function of anisotropy in several size regimes reveal that the maximal SAR would be obtained at the single-domain ferromagnetic size regime [17]. So, producing high-moment magnetic nanoparticles in this range is of high value from technical and clinical aspects.

There are several works dealing with the magnetic properties of iron compounds including its oxides and alloys for use in hyperthermia treatment [14-19]. For example, Hong et al. have synthesized Fe_3_O_4_ nanoparticles using co-precipitation method and have shown that magnetic fluids of Fe_3_O_4_ nanoparticles which are coated with a surfactant bilayer feature high stability even after diluting and autoclaving and therefore are suitable for being used in magnetic hyperthermia treatment [16].

Among iron compounds, FeCo alloys are known to exhibit the highest magnetic properties. Iron and cobalt are both near the peak of the Slater-Pauling curve and have maximum saturation magnetization when combined together. Fe_0.7_Co_0.3_ has the highest saturation magnetization among all magnetic alloys [20].

Till now, several methods have been used to synthesize FeCo alloy nanoparticles which include arc discharge [21], polyol [2], hydrothermal process [6], RAPET [7], thermal decomposition [9], wet chemical methods [10,11], and co-precipitation [13]. The morphology and size distribution of as-synthesized nanoparticles are not well controlled in most of these processes. To attain the best properties for magnetic hyperthermia, the size distribution is an effective parameter. Researches show the loss of SAR due to the size distribution of nanoparticles. So, employing a method capable of producing monodisperse nanoparticles is very important. Also, stabilizing the magnetic fluid to prevent the agglomeration of nanoparticles is necessary so that the magnetic properties of the fluid would not change with time.

Among all synthetic routes, the microemulsion technique has the capability of controlling the shape, size, and size distribution of nanoparticles [21]. In this process, the precipitation of nanoparticles takes place inside nanocages called micelles. The micelle is in the form of sphere or cylinder of oil in water (normal micelle) or water in oil (reverse micelle) which is surrounded by a layer of surfactant molecules [22]. The morphology of micelles depends on the type of the surfactant and water-to-surfactant molar ratio (*R*). The technique could be used to synthesize mineral [23] or organic compounds [24] inside the nanoreactors. Metals or metal oxides could be produced by reduction of their mineral salts with a reductant such as borohydride.

In the present research, the shape- and size-controlled synthesis of iron-cobalt alloy nanoparticles was carried out in reverse micelles of water in hexanol, and the magnetic properties of synthesized nanoparticles were studied. Then, the magnetic fluids of each series of nanoparticles were prepared, and the stability and inductive properties of FeCo nanofluids were studied. Finally, the mechanisms of heat generation were discussed based on experimental results and theoretical models.

## Methods

Iron(III) chloride hexahydrate (FeCl_3_ · 6H_2_O (%99+)), cobalt(II) sulfate heptahydrate (Co(SO_4_) · 7H_2_O (%99+)), 1-hexanol, sodium borohydride (NaBH_4_ (%99+)), and cetyltrimethylammonium bromide (CTAB) were purchased from MERCK chemicals (Saadat Abad, Tehran, Iran) and used as received with no further purification. High-purity nitrogen gas (%99.99+) was used to provide an oxygen-free environment during the synthesis procedure. Microemulsion 1 (ME1) and microemulsion 2 (ME2) were prepared on the basis of ternary phase diagram of water/CTAB/hexanol which is described elsewhere [25].

Fe_0.7_Co_0.3_ alloy nanoparticles were prepared by mixing equal volumes of ME1 and ME2 containing metal salts and precipitating agent, respectively. The [NaBH_4_]/[metal salts] molar ratio was kept at 2 with metal salt concentration of 0.1 M. First, ME1 was transferred into a three-necked round-bottomed flask and then ME2 was added drop by drop with vigorous stirring of ME1 under N_2_ atmosphere. Black precipitates of FeCo alloy nanoparticles appeared immediately after mixing of the two microemulsions. After 5 min of reaction, the synthesized nanoparticles were magnetically separated using a strong neodymium magnet, and the supernatant was decanted. Then, the nanoparticles were washed with acetone, ethanol, and chloroform several times to remove all residual elements and compounds. Some of the as-synthesized powders were annealed in a tube furnace at 623 and 823 K for 10 min under H_2_ atmosphere.

To maintain a magnetic fluid with stable dispersion, FeCo nanoparticles were dispersed in a vigorously stirring solution of CTAB (2 gr)/1-butanol (2 ml) in deionized water for 1 h under an inert atmosphere.

**Figure 1:**
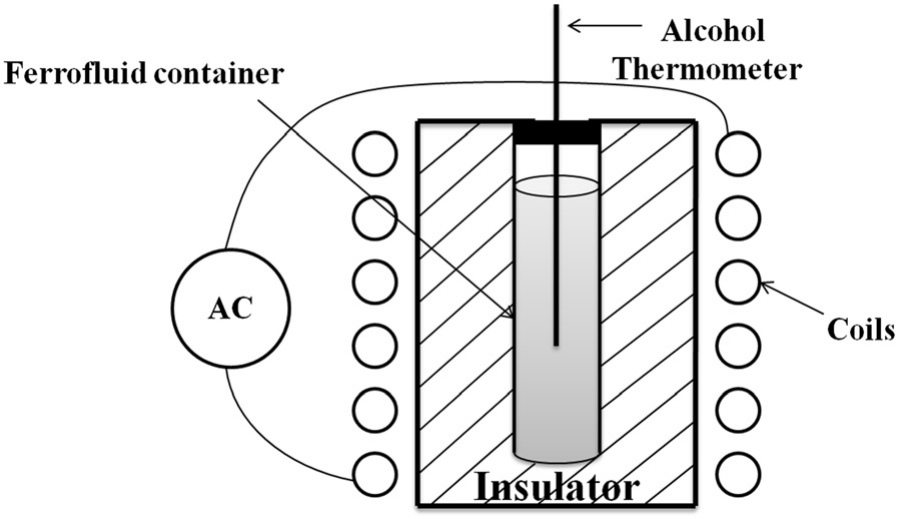
Schematic representation of the experimental setup for inspecting the inductive properties of magnetic fluids.

Characterization of samples was done using X-ray diffraction (XRD) (PANalytical X'Pert Pro MPD (PANalytical B.V., Almelo, The Netherlands) with Cu kα radiation), transmission electron microscopy (TEM) (ZEISS EM10-C (Carl Zeiss AG, Oberkochen, Germany) at 100 kV), and high-resolution transmission electron microscopy (HRTEM) (JEOL JEM-2100 (JEOL Ltd., Tokyo, Japan) at 200 kV). Elemental analysis was done using an energy-dispersive spectroscopy (EDS) detector attached to the HRTEM. The magnetic properties of samples were analyzed using a vibrating sample magnetometer (VSM). The stability of the magnetic fluids was investigated using a Gouy magnetic susceptibility balance instrument (MSB-MK1) at various nanoparticle sizes and concentrations. Finally, the inductive heating properties of magnetic fluids with 0.8% volume fraction of nanoparticles were investigated using an AC magnetic field generator with *H* = 20 kA m^-1^ and *f* = 120 kHz. The schematic representation of the used apparatus is shown in Figure 1. The samples and process conditions are summarized in Table 1.

**Table 1:** Samples and process condition

**Sample**	**Water/surfactant molar ratio (*R*)**	***T *** **(K)**
W1	7	300
W2	14	300
W3	20	300
W4	27	300
A1	-	623
A2	-	823

## Results and discussion

### Structural characterization

**Figure 2:**
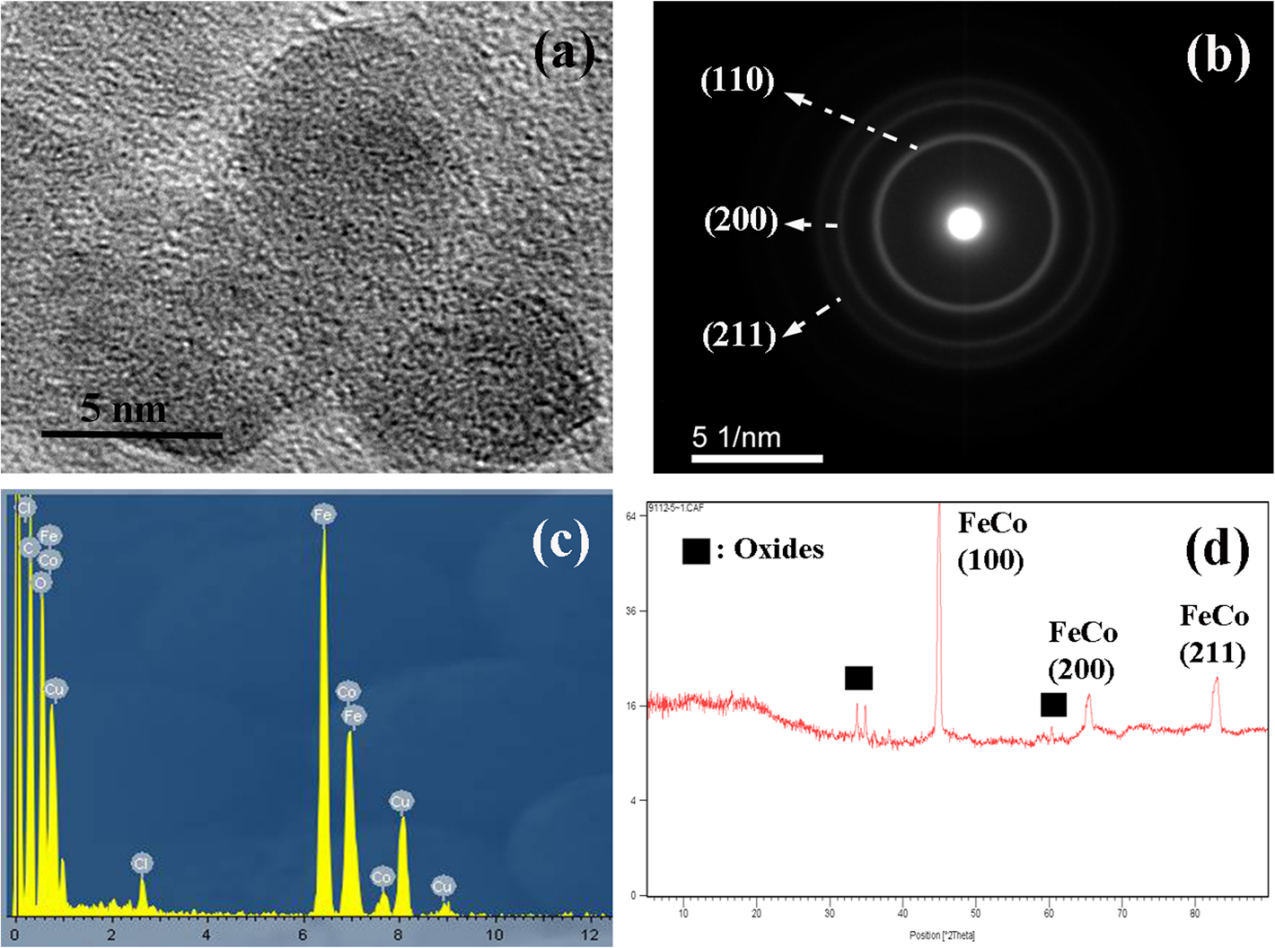
**Characterization of the W4 sample. (a)** HRTEM micrograph. **(b)** Selected area diffraction pattern. **(c)** EDS spectrum. **(d)** XRD patterns.

Figure 2a shows the high-resolution TEM image of the W4 sample. The bad crystallinity of as-synthesized nanoparticles is due to fast borohydride reduction which prevents lattice planes from being arranged in a complete crystalline manner. Electron beam and X-ray diffraction patterns (Figure 2b,d) indicate the formation of a bcc-structured iron-cobalt alloy. Also, a small quantity of CoFe_2_O_4_ (at 2*θ* = 35.4°, 62.4°) is observed due to partial oxidation of the sample due to the exposure of nanoparticles to air. This also is confirmed by the presence of an oxygen peak in the EDS spectrum in Figure 2c. Therefore, it could be inferred that a thin oxide film has been formed around the synthesized nanoparticles. The EDS analysis also shows Fe and Co peaks in which the Fe peak is sharper, indicating higher content of Fe than Co.

**Figure 3:**
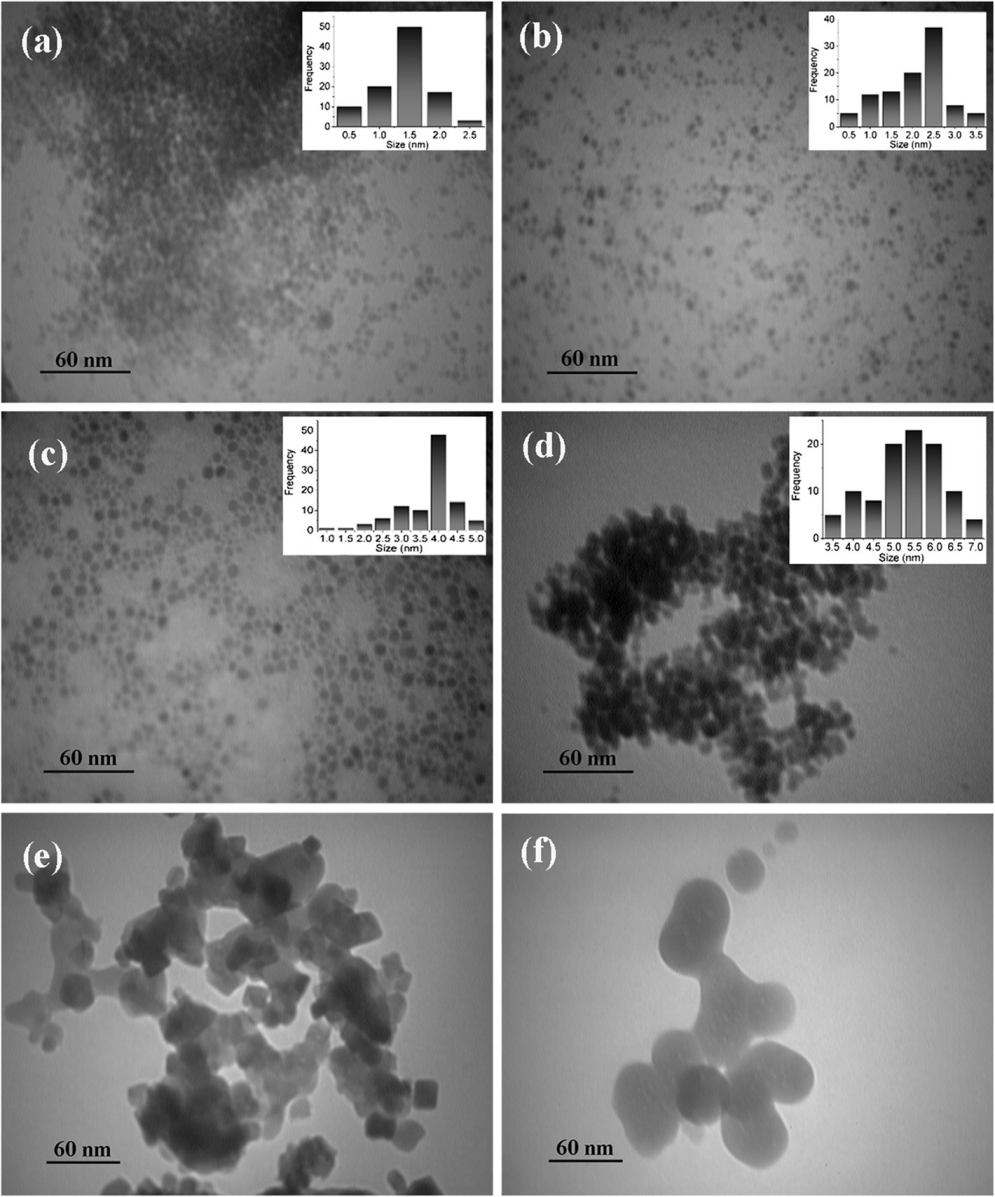
**TEM micrographs of as-synthesized nanoparticles and corresponding size distributions. (a)** W1, **(b)** W2, **(c)** W3, **(d)** W4, **(e)** A1 (W4 annealed at 623 K) for 10 min, and **(f)** A2 (W4 annealed at 823 K) for 10 min.

Figure 3 shows the effect of water-to-surfactant molar ratio (*R*) on the morphology, size, and size distribution of as-synthesized nanoparticles. The mean size and size distribution for each specimen were determined by inspecting about 50 TEM micrographs. It is evident that all samples have spherical shape due to the nature of the oil-surfactant-water system used.

Figure 3 shows an expected increase in the mean size of nanoparticles with *R* because as the *R* value increases, the relative amount of water increases and a larger micelle would be obtained; thus, the limiting stability of nanoreactors decreases, leading to larger nanoparticles. It should be noted that at *R* > 27, the transparent microemulsion could not form, indicating that the maximum available *R* for this ternary system is 27. This means that with the ternary system of water/CTAB/hexanol, the maximum achievable size for the FeCo nanoparticle is about 7 nm.

Figure 3e,f shows TEM images of the W3 sample annealed at 623 and 823 K for 10 min, respectively. It is seen that nanoparticles have grown by the fusion of smaller nanoparticles to the mean sizes of 36 and 60 nm, respectively.

**Figure 4:**
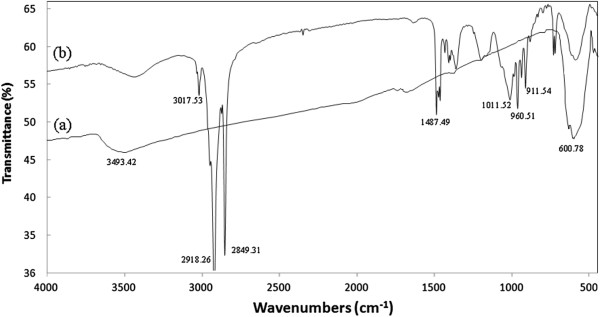
FTIR spectra for (a) as-synthesized FeCo nanoparticles and (b) CTAB/1-butanol-functionalized FeCo nanoparticles.

The surface chemical structure of nanoparticles was studied using Fourier transform infrared (FTIR) spectroscopy. Figure 4a shows the FTIR spectra for as-synthesized FeCo nanoparticles. The broad but intense peak at 600.78 cm^-1^ is the vibration of M_T_-O-M_O_ bonds corresponding to the bond between oxygen and atoms (M) at tetrahedral and octahedral sites in the spinel structure of CoFe_2_O_4_[26]. The broad peak at 3,493.42 cm^-1^ is characteristic of O-H bonds which are present on the surface of FeCo nanoparticles. In Figure 4b, the peaks between 900 and 1,000 cm^-1^ are due to the wagging of C-N bonds in CTAB molecules [27]. Also, the broad peak at 1,011.52 is from the C-O vibration in 1-butanol. The series of intense peaks at 1,487 cm^-1^ and 2,800 to 3,000 cm^-1^ are related to bending and stretching of C-H bonds in 1-butanol and the hydrophobic chain of CTAB. The results confirm that the partially oxidized FeCo nanoparticles are successfully functionalized with a bilayer of CTAB/1-butanol.

### Magnetic properties of FeCo nanoparticles

**Figure 5:**
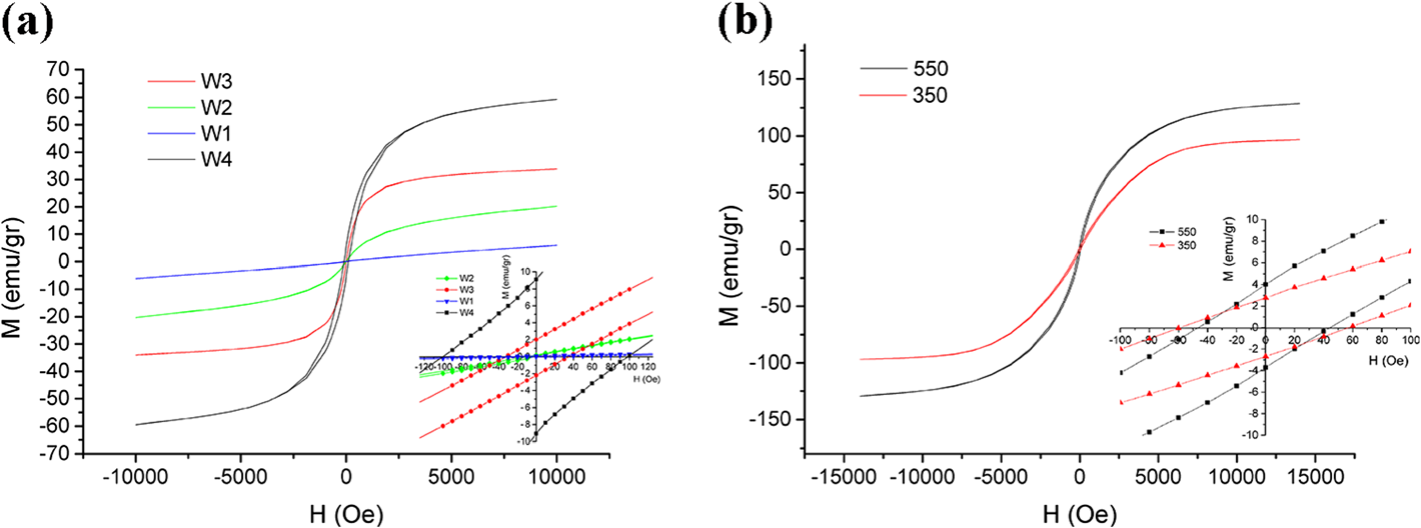
Hysteresis curves for (a) as-synthesized nanoparticles and (b) annealed nanoparticles.

Figure 5a,b shows hysteresis curves for as-synthesized and annealed samples. Magnetic properties of as-synthesized nanoparticles along with their mean particle sizes are shown in Table 2.

**Table 2:** Magnetic properties of as-synthesized nanoparticles

**Sample**	**Water/surfactant molar ratio (*R*)**	**Mean size (nm)**	***M***_**s **_**(emu/g)**	***M***_**r **_**(emu/g)**	***H***_**c **_**(Oe)**
W1	7	2	6	0	0
W2	14	2.5	20	0	2
W3	20	4	33	2	40
W4	27	5.5	60	9	100
A1	-	36	90	2.5	60
A2	-	60	125	4	40

**Figure 6:**
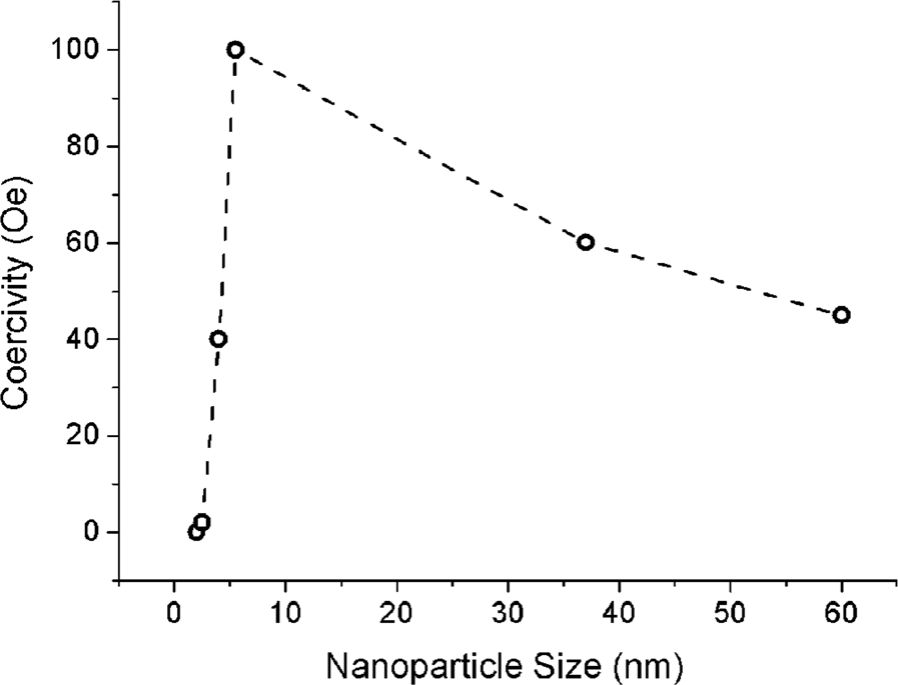
Coercivity as a function of particle size.

It can be seen that the magnetic properties of as-synthesized FeCo nanoparticles are well controlled by the *R* value. By decreasing the nanoparticle size, the atomic orbitals overlap due to the bond length contraction [28] and electron spins become disordered because of the increasing number of dangling bonds at the nanoparticle surface [29], and therefore, the saturation magnetization decreases. Figure 6 shows the change in *H*_c_ with particle size. The plot has a maximum at the size of 5.5 nm which is near the single-domain-multi-domain boundary at which the mechanism of magnetization changes from coherent reversal of a macro spin to the domain wall motion [20]. In fact, below a certain value of nanoparticle size, *H*_c_ decreases rapidly.

The coercivity change in Figure 6 confirms that as-synthesized nanoparticles are in the single-domain range. For single-domain nanoparticles, the coercivity is proportional to *d*^6^[30]:

(3) $$H_{c}=\frac{\alpha_{1}K^{4}}{AJ_{s}}d^{6}\propto d^{6}$$

where *α*_1_ is a constant, *A* represents the exchange stiffness, *K* is the effective anisotropy constant, *J*_s_ is the exchange energy density, and *d* is the nanoparticle size. The experimental values of *H*_c_ are in good agreement with this theoretical expression, indicating that as-synthesized nanoparticles are in the single-domain size range. However, for multi-domain nanoparticles showing ferromagnetic behavior, the domain wall motion is the main mechanism of the magnetic reversal. In this size range, the pinning of the domain walls to lattice obstacles such as grain boundaries is the main source of the coercivity. The theory predicts [30]

(4) $$H_{c}=\frac{\alpha_{2}{\left(\mathrm{AK}\right)}^{1/2}}{J_{s}d}\propto d^{-1}$$

where *α*_2_ is another constant. The results obtained for A1 and A2 samples match the above proportion, indicating that annealed nanoparticles are in the multi-domain size range. The boundary between these two cases in Equations 3 and 4 is the ferromagnetic exchange length $d_{\mathrm{ex}}=\sqrt{\left(A/K\right)}$. For Fe_0.7_Co_0.3_, the values of *A* and *K* are 2.6 × 10^-12^ (J m^-1^) [31] and 4.2 × 10^4^ (J m^-3^) [18], respectively, resulting in the exchange length of 7.86 nm. Below this size, *H*_c_ will decrease rapidly as the particle size decreases. When *H*_c_ reaches zero, nanoparticles exhibit superparamagnetic properties with a null hysteresis area as observed in the W1 sample.

### Stability and inductive properties of FeCo magnetic fluids

#### Stability of FeCo magnetic fluids

**Figure 7:**
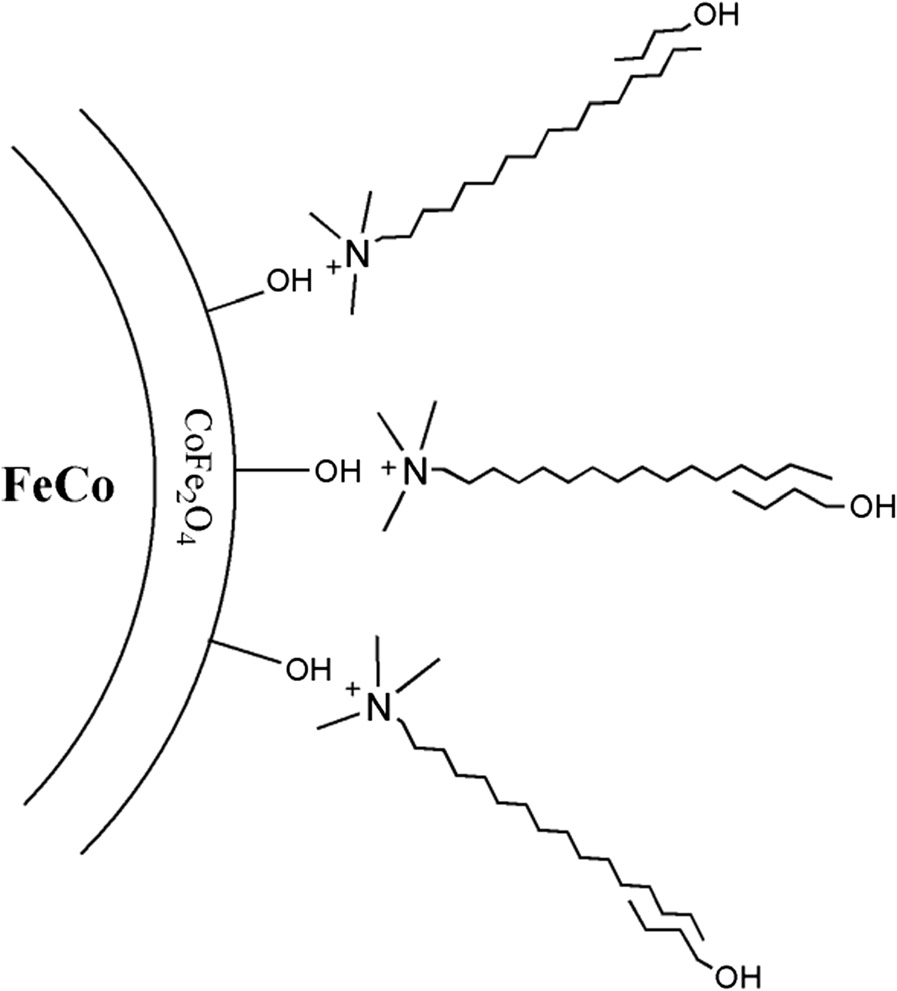
Schematic representation of CTAB/1-butanol bilayer on the surface of FeCo nanoparticles.

The CTAB coating on the surface of FeCo nanoparticles is an antiseptic agent against bacteria and fungi and is used as a buffer solution for the extraction of DNA. It has been used as a stabilizing agent for magnetite nanoparticles in MRI [32]. CTAB is a positively charged cationic surfactant. By considering the isoelectric point (pH_IEP_) of CoFe_2_O_4_ which is about 6.9 [33], it could be inferred that at pH = 7, the surface of nanoparticles is negatively charged and therefore is easily bound to the cationic head of CTAB via electrostatic interactions similar to what was reported for tetramethylammonium hydroxide (TMAOH) on the surface of Fe-based magnetic nanoparticles [27,34]. Also, 1-butanol with a hydrophilic hydroxyl head has an aliphatic chain which is compatible with the long molecular chain structure of CTAB. Therefore, CTAB/1-butanol could form a bilayer around FeCo nanoparticles which makes them stable in the fluid. Figure 7 shows the schematic representation of the CTAB/1-butanol bilayer formation on the surface of FeCo nanoparticles.

#### Effect of nanoparticle size

**Figure 8:**
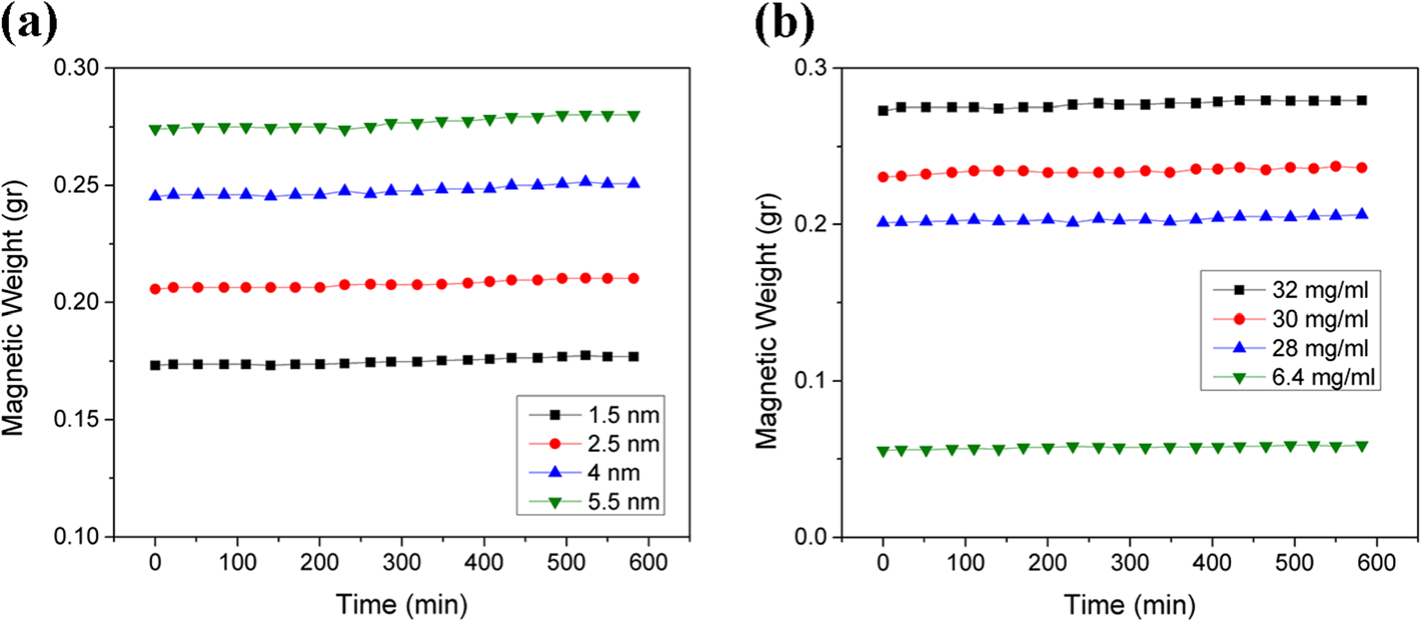
**Stability of functionalized FeCo nanoparticles. (a)** Effect of nanoparticle size. **(b)** Effect of nanoparticle concentration.

The stability of the magnetic fluids was studied at each nanoparticle size by inspecting the weight change of magnetic fluids with respect to time at the constant magnetic field of 20 mT which is normally used in hyperthermia treatments [17]. Figure 8a shows the stability of magnetic fluids for various nanoparticle sizes at the concentration of 32 mg/ml. As observed, all samples exhibit good stability due to the presence of the CTAB/1-butanol bilayer on the surface of FeCo nanoparticles. It is seen that the magnetic weight changes from 0.003 gr for magnetic fluid of 1.5-nm nanoparticles to 0.006 gr for that of 5.5-nm nanoparticles.

There are two reasons based on the shape of agglomerates: (1) By increasing the nanoparticle size, the bulk magnetic properties dominate the surface properties, the portion of the magnetic dead layer on the nanoparticle surface decreases, and simultaneously the magnetic moment of nanoparticles increases [29]; therefore, the magnetic dipoles may be aligned and a chain-like aggregation of nanoparticles could form, leading to the precipitation of nanoparticles [35]. (2) By increasing the nanoparticle size at a fixed concentration, the increased proximity of surface atoms from adjacent nanoparticles results in inter-particle exchange interactions, leading to the formation of a collective state which in the case of randomly distributed nanoparticles is very similar to a spin glass [35]. Therefore, the net magnetic moment of the agglomerate will decrease, and the applied field of 20 mT would not be sufficient to suspend the aggregation; therefore, the precipitation occurs. Table 3 shows the susceptibility of magnetic fluids of various nanoparticle sizes at 32 mg/ml concentration.

**Table 3:** Magnetic susceptibility of prepared fluids with various nanoparticle sizes at 32 mg/ml concentration

**Nanoparticle mean size (nm)**	**Susceptibility (*χ*) × 10**^**-5**^
1.5	1.46
2.5	3.94
4	6.73
5.5	10.74

#### Effect of magnetic fluid concentration

To study the effect of nanoparticle concentration on the stability of magnetic fluids, W4 nanoparticles which have the largest mean size among all samples were used to prepare magnetic fluids with different concentrations. Figure 8b shows the change of magnetic weight with time; for 32, 30, and 28 mg/ml, the magnetic weight reduces to 0.006, 0.006, and 0.005 gr, respectively. It is seen that the higher the concentration of nanoparticles, the greater the decrease of magnetic weight. In fact, at higher concentrations, nanoparticles are in lower spatial distances, and therefore, the probability of precipitation is higher based on the mechanisms described in the previous section.

Also, the effect of dilution was investigated at the ratio of 1:5 by reducing the nanoparticle concentration from 32 to 6.4 mg/ml. It is seen that the magnetic fluid is stable even after being diluted since the reduction of magnetic weight is about 0.002 gr. This is in line with the results reported by Hong et al. on the stability of Fe_3_O_4_ nanofluids [16]. As they reported for magnetite nanoparticles, the reason is that the surfactant bilayer could not be destroyed when the magnetic fluid is diluted.

#### SAR measurements

**Figure 9:**
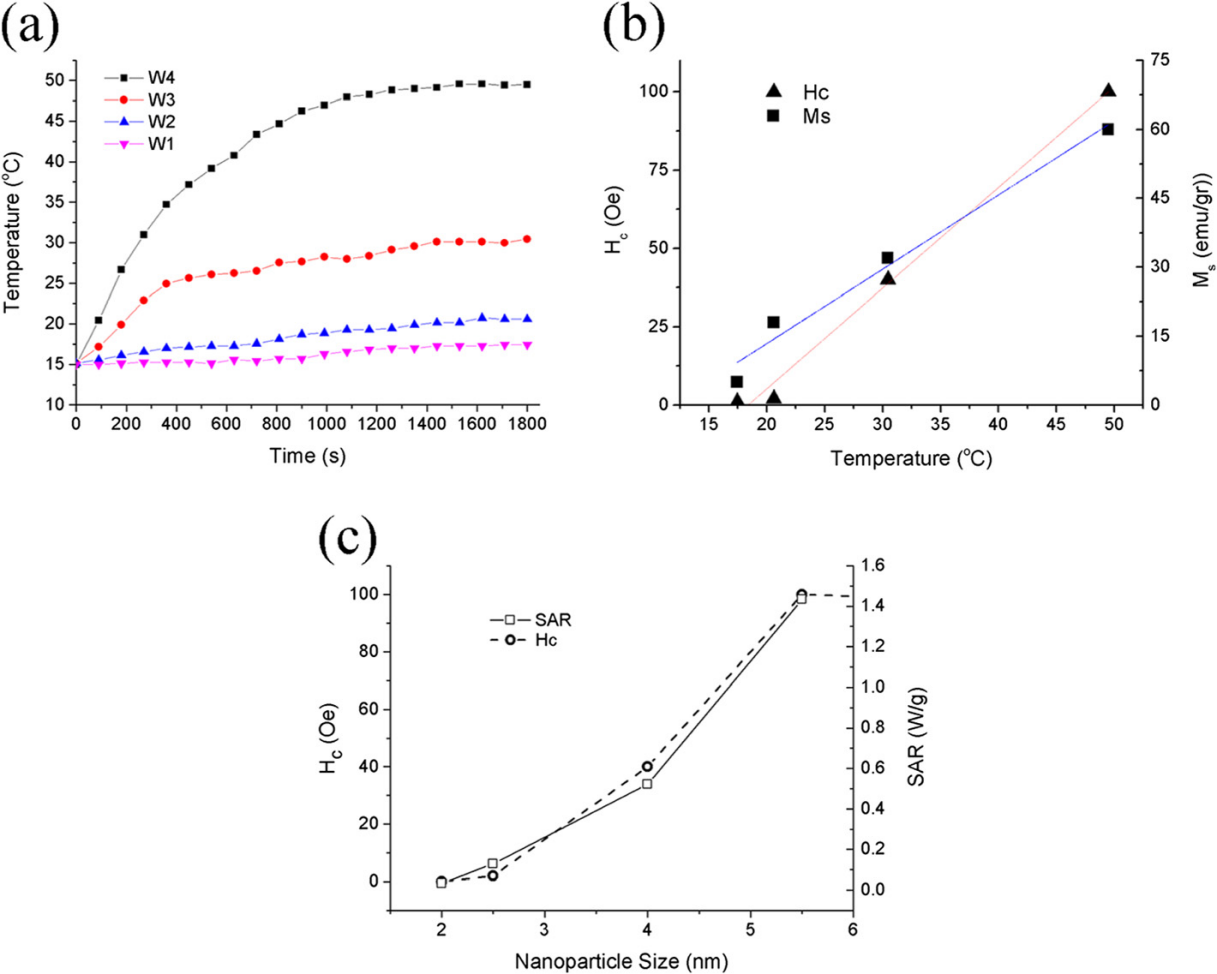
**Inductive properties of FeCo magnetic nanofluids. (a)** Temperature rise of magnetic fluid as a function of time under AC magnetic field at various nanoparticle sizes (*f* = 120 kHz). **(b)** Obtained temperature as a function of *H*_c_ and *M*_s_. **(c)** Matched dependence of SAR and *H*_c_ on the nanoparticle size.

Figure 9a shows the evolution of temperature for magnetic fluids containing W1 to W4 nanoparticles after switching on the magnetic field at fixed values of *H* = 20 kA m^-1^ and *f* = 120 kHz. It should be noted that A1 and A2 samples were only used to study the dependence of coercivity on the nanoparticle size and were not capable of being used for hyperthermia treatment because of their large size and also the agglomeration of nanoparticles which both increase the probability of their precipitation and consequent blockage of blood vessels, making the chance for the phagocytosis system to detect them. The corresponding SAR values of as-synthesized samples could be calculated by the formula [36]

(5) $$\mathrm{SAR}={\left(\frac{dT}{dt}\right)}_{0}\frac{C_{w}m_{w}+C_{\mathrm{FeCo}}m_{\mathrm{FeCo}}}{C_{\mathrm{FeCo}}m_{\mathrm{FeCo}}}$$

where (d*T*/d*t*)_0_ is the initial slope of the *T*-*t* curve, *C*_w_ is the specific heat of water, *C*_FeCo_ is the specific heat of FeCo nanoparticles, *m*_w_ is the mass fraction of water in the fluid, and *m*_FeCo_ is the mass fraction of FeCo nanoparticles in the fluid. The (d*T*/d*t*)_0_ values were calculated by differentiating the second-order polynomial fit of *T*-*t* curves at *t* = 0 where *C*_w_ and *C*_FeCo_ are 4,190 J (kg K)^-1^[36] and 120.11 J (kg K)^-1^[37].

As seen from Figure 9a, the temperature increases with time and saturates after 1,800 s has elapsed, showing a behavior predicted by the Box-Lucas Equation *T*(*t*) = *A*(1 - e^-Bt^) which is often used for describing the alternating magnetic field properties of magnetic nanoparticles [36].

It is also seen that the generated heat and specific absorption rate of nanoparticles increase with increasing of the nanoparticle size such that for the W4 sample with a mean size of 5.5 nm, a temperature rise of 23 K was obtained compared with that of the W3, W2, and W1 samples (11, 4, and 2.5 K) (Table 4). In order to destroy tumor cells, the local temperature should be raised between 5 and 9 K [15]. Thus, at this frequency which is the conventional clinical frequency, only W4 and W3 samples could be used as suitable thermoseeds with corresponding temperature rises of 23 and 11 K.

**Table 4:** Inductive properties of prepared magnetic fluids

**Sample**	**Mean size (nm)**	**Temp. rise (°C)**	**SAR (W g**^**-1**^**) (experimental)**	**SAR (W g**^**-1**^**) (SW model)**	**SAR (W g**^**-1**^**) (LRT)**
W1	2	2.5	0.032	-	-
W2	2.5	4	0.129	-	-
W3	4	11	0.522	165	≈0.84 × 10^-3^
W4	5.5	23	1.434	540	≈11 × 10^-3^

Figure 9b indicates a direct relation of temperature rise with *H*_c_ and *M*_s_ which means that the generated heat increases by enhancing the hysteresis area, showing an important contribution of hysteresis loss to the generated heat.

Also, as observed from Figure 9c, the tendency of SAR to change with particle size is perfectly matched to the tendency of *H*_c_ values. This is due to the fact that there is a central parameter which determines both the coercivity and maximum achievable SAR and also controls the influence of the size distribution of nanoparticles on the SAR [17]. This parameter is the anisotropy of nanoparticles which has the following optimum value that results in the largest possible SAR for random orientation nanoparticles [17]:

(6) $$K_{\mathrm{opt}}=0.84\left(\mu_{0}H_{\max}M_{s}\right)$$

**Figure 10:**
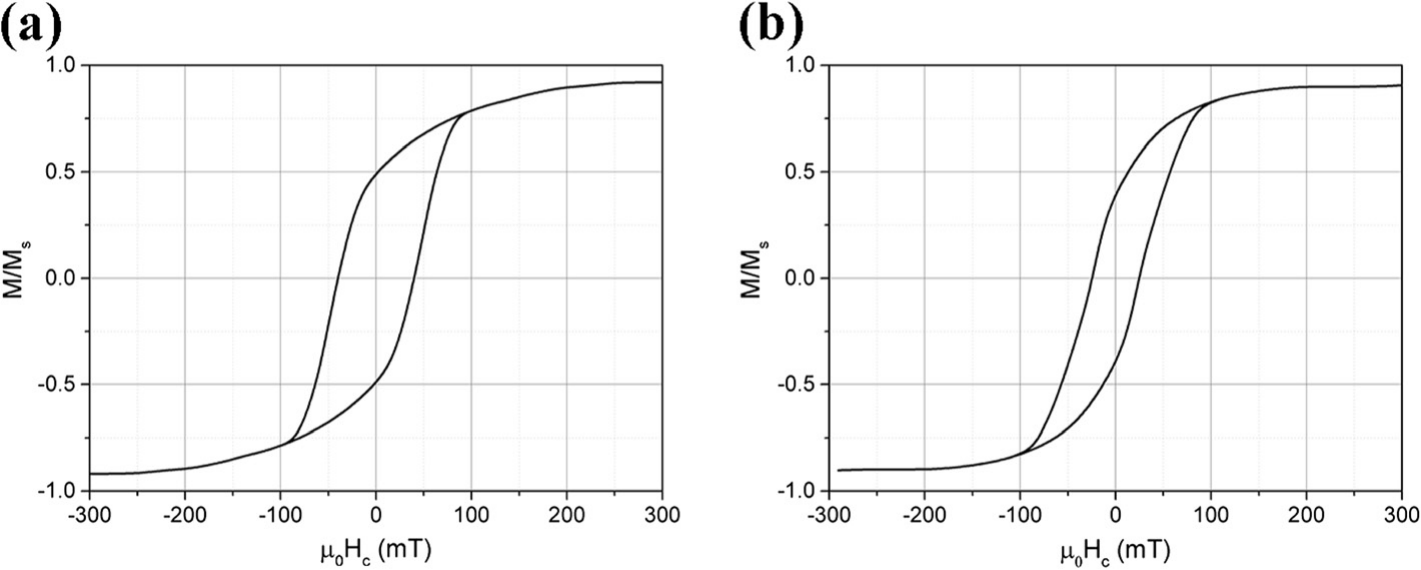
**Hysteresis curves of the colloidal solutions at *T*** **= 2 K. (a)** W4 and **(b)** W3.

Considering *H*_max_ = 20 (kA m^-1^), the value of *K*_opt_ for W4 and W3 samples will be 1.05 × 10^5^ (J m^-3^) and 5.78 × 10^4^ (J m^-3^), respectively. Figure 10 shows hysteresis curves for W4 and W3 samples at 2 K.

For random orientation nanoparticles, the frequency and temperature dependence of the coercive field is described by the following equation [18]:

(7) $$\mu_{0}H_{c}=\frac{2K}{\rho M_{S}}\left[0.479-0.81{\left(\frac{k_{B}T_{1}}{2\mathrm{KV}}\left(\ln\frac{1}{f\tau_{0}}\right)\right)}^{\frac{3}{4}}\right]$$

where *ρ* = 8,300 (kg m^-3^) is the density of FeCo alloy, *k*_B_ is the Boltzmann constant, *V* is the volume of nanoparticles, *f* = 5.5 × 10^-4^ (Hz) is the measurement frequency, and *τ*_0_ = 10^-10^ (s) is the intrawell relaxation time. Therefore, by considering the values of *μ*_0_*H*_c_ from Figure 10a,b, the anisotropy constants for W4 and W3 are calculated to be 4.1 × 10^4^ (J m^-3^) and 6.64 × 10^4^ (J m^-3^), respectively. Comparing the anisotropy values obtained from magnetic measurements with the optimum anisotropies from Equation 6 reveals that for the W3 sample, these two values are very close together, indicating that the maximum generated heat for this sample is around that which we obtained experimentally, but for the W4 sample, the optimum anisotropy is about 2.5 times greater than the experimental value. As the result of this deviation from the optimum value in the W4 sample (which also exhibits broader size distribution than W3 sample (see Figure 3)), the detrimental effect of nanoparticle size distribution makes the maximum achievable SAR decrease. As noted by Carrey et al., the particle size distribution has a negative effect on the maximum achievable SAR, and the anisotropy controls this effect [17].

As mentioned earlier, superparamagnetic W1 and W2 samples are useless for hyperthermia treatment, but W3 and W4 samples are in the single-domain ferromagnetic size regime and capable for use in hyperthermia. Considering the domain of validity of SW and LRT models which are *μ*_0_*H*_max_ > 2 *μ*_0_*H*_c_ and *ξ* = (*μ*_0_*M*_s_*VH*_max_)/(*k*_B_*T*) < 1, respectively, we could apply both SW and LRT models to both W3 and W4 samples to discuss the involved mechanisms in the generation of heat. Applying the model proposed by Stoner-Wohlfarth for random orientation nanoparticles we have (as seen in Equation 2)

$$A=2\mu_{0}H_{C,\left(T=0\;K\right)}M_{s}$$

Assuming *f* = 120 kHz, the corresponding SARs for W4 and W3 samples are 540 and 165 (W g^-1^), respectively. If we apply the LRT model instead, by considering *τ*_R_ = *τ*_N_ = *τ*_0_exp(*K*_eff_*V*/*k*_B_*T*), the values of SAR could be calculated from Equation 1 as seen in Table 4.

The comparative study between experimental and theoretical values of SAR indicates the following: (a) The experimental values are between pure hysteresis (SW model) and pure relaxation (LRT) which means that both loss mechanisms are involved. (b) Assuming the maximum contribution of relaxation to the total loss, for the W3 sample, the contribution of relaxation to the total SAR is 0.16% and the remaining SAR belongs to the hysteresis (99.84%), and for the W4 sample, the corresponding values are 0.76% and 99.24%, respectively, indicating that hysteresis is a more effective mechanism in producing of heat. (c) W4 ferromagnetic nanoparticles exhibit higher experimental and theoretical SAR compared with other samples. As noted by Lacroix et al. [18], given the weak magnetic fields in hyperthermia treatment, the maximum SAR would be obtained for soft ferromagnetic nanoparticles or the nanoparticles near the superparamagnetic transition. This is consistent with our experimental results.

## Conclusions

Size-controlled synthesis of FeCo nanoparticles was done using microemulsion method. It was observed that by increasing the water-to-surfactant molar ratio, the nanoparticles become larger. The maximum size of nanoparticles in the ternary system of water/CTAB/hexanol is about 7 nm. Size dependency of magnetic properties including *M*_s_ and *H*_c_ was investigated. The observed increase in *M*_s_ with size is due to disappearance of the magnetic dead layer in larger nanoparticles. However, the observed change in coercivity with size is due to transition between various size regimes and consequently the magnetization reversal mechanisms. The nanoparticles were stabilized using a CTAB/1-butanol bilayer. The stability of nanoparticles was studied at various nanoparticle sizes and concentrations. Results show that by increasing the nanoparticle size or concentration, the stability of the magnetic fluid decreases due to magnetic interaction and consequent aggregation of nanoparticles.

The inductive properties of nanoparticles such as temperature rise and specific absorption rate were evaluated at various nanoparticle sizes and were observed to have direct relation with the size of nanoparticles. Both *H*_c_ and SAR show similar tendencies of changing with particle size. The reason lies in anisotropy as a central parameter controlling both *H*_c_ and SAR.

Only W4 and W3 ferromagnetic nanoparticles are found to be capable of being used in hyperthermia treatment which passed the minimum temperature rise of 5°C to 9°C. The comparison of experimental results with those of Stoner-Wohlfarth and LRT models shows that hysteresis and relaxation mechanisms are both involved in the generation of heat, but the contribution of hysteresis is far greater than relaxation.

## Competing interests

The authors declare that they have no competing interests.

## Authors' contributions

SSSA carried out the nanoparticle synthesis; conducted FTIR, XRD, and nanofluid stability experiments and magnetic studies; and drafted the manuscript. AS carried out TEM characterization of samples and revised the drafted manuscript to prepare it for submission. Both authors read and approved the final manuscript.
